# Exploring the dynamics and future projections of land use land cover changes by exploiting geospatial techniques; A case study of the Kabul River Basin

**DOI:** 10.1016/j.heliyon.2024.e39020

**Published:** 2024-10-05

**Authors:** Rahmatullah Wahdatyar, Muhammad Fahim Khokhar, Shakil Ahmad, Mohammad Uzair Rahil, Mohammad Ajmal Stanikzai, Junaid Aziz Khan, Kamran

**Affiliations:** a School of Civil and Environmental Engineering (SCEE), National University of Sciences and Technology (NUST), Sector H-12, Islamabad, 44000, Pakistan; b Department of Water Resources and Environmental Engineering, Nangarhar University, Afghanistan

**Keywords:** Random forest classifier, Cellular automata, LULC, Kabul river basin, MOLUSCE plugin

## Abstract

Global land cover change has caused significant environmental degradation and biodiversity loss. It affects ecosystem functions, livelihoods, and climate variation and has drawn substantial attention in recent decades. In the Kabul River Basin (KRB), there are limited studies on the historical Land Use/Land Cover (LULC) pattern, transition, intensity and future perspective. Therefore, this study aims to investigate long-term LULC changes and major drivers of LULC in the KRB over the past thirty years (1990–2020) and then to project the future LULC pattern for the years 2030, 2040 and 2050. Landsat Imageries of (1990–2020) were used as input data by utilizing the Random Forest Classifier algorithm (RF) in the Google Earth Engine (GEE) to classify the LULC. The LULC was then projected for the future, using the Cellular Automata Markov Chain Model (CA-MCM). The results demonstrated drastic LULC changes, controlled primarily by urbanization and agriculture expansion, which expanded from 467 Km^2^ (0.7 %) to 2312 km^2^ (3.4 %) and 6528 km^2^ (9.6 %) to 10812 (15.9 %), between 1990 and 2020. In contrast, bare land decreased from 70606 km^2^ (82.1 %) to 48212 km^2^ (70.9 %) between 1990 and 2020. In addition, the study depicts that the expansion in built-up and vegetation areas in the KRB during the study period were at the utilization of bare land. Future LULC predictions indicated that between 2020 and 2050, bare land would trend downward from 48212 km^2^ (70.9 %) to 46172 km^2^ (67.9 %), while vegetation and built-up areas would trend upward from 2312 km^2^ (3.4 %) to 3640 km^2^ (5.3 %), 10812 km^2^ (15.9 %) to 11622 km^2^ (17.1 %), and water bodies and snowcover would slightly vary from 1.2 % to 0.9 % and 7.9 %–9.0 %. In addition, the results of LULC dynamics reveal a significant strong positive correlation between population and built, as well as population and vegetation. Conversely, there is a strong negative correlation between population and bare land. Our results provide precise insights on LULC patterns and trends in the KRB, which could be employed to design a sustainable framework for land use and ecosystem protection.

## Highlights

• First attempt to investigate and project long term LULC in Kabul River Basin (KRB).
• Built-up area has been drastically increased followed by vegetation.
• Substantial decrease occurred in bare land and mainly transitioned to agriculture.
• Study guides about future environmental challenges in transboundary river basin.

## Graphical abstract

**Figure:**
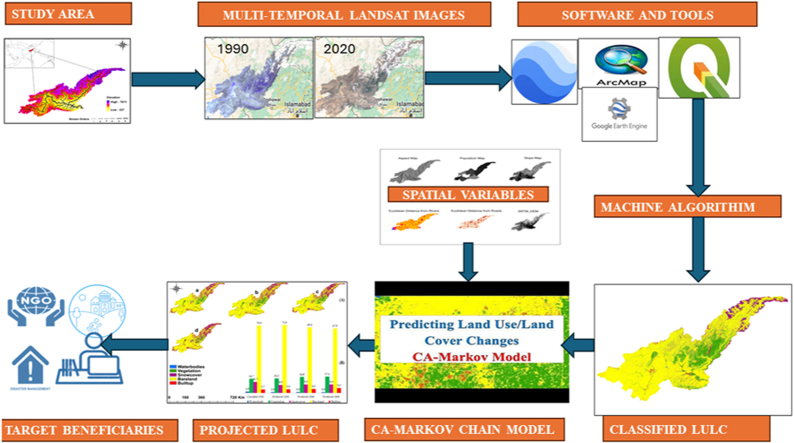

## 1 Introduction

Land use/land cover change (LUCC) is a global phenomenon that has substantial environmental implications and biodiversity loss [1]. The primary consequence of LULCC is ecosystem degradation, which increase surface runoff and urban heat, influences various climate change components, and decreases groundwater levels [2,3]. Over the past four decades, the increase in LULCC has accelerated the environmental crisis, disturbing ecosystem processes that are crucial for human population [4]. Several factors affecting, LULCC like politics, time, scale, social and culture and economics factors [5,6]. Land Use/Land Cover (LULC) alterations have been identified as important drivers of environmental changes at all geographical and temporal dimensions and is used for monitoring the ecological changes [[7], [8], [9]]. Therefore, it is essential for urban planners, government agencies, hydrologists, and environmentalists to monitor and identify changes in land use and cover. Additionally, projecting future changes in land cover and usage offers a strategic framework for putting long-term improvements in resource planning and management into practice [10]. Hence, having dependable and up-to-date land cover data is essential for the effectiveness of monitoring, planning, and management initiatives [11]. The utilization of RS and GIS information is a viable approach for investigating changes in LULC. These tools are highly capable and efficient experiencing growing utilization in evaluating urbanization and conducting change-detection analyses [12].

GIS present a useful platform of collecting, analyzing, storing, and providing important digital data for investigating changes in LULC [13]. However, producing large-scale LULC maps is difficult, due to a variety of factors including heterogeneity, land surface's spectral complexity, and the limitation of the required hardware and software. For instance, multi-temporal imagery is frequently needed to differentiate different kinds of LULC, however this approach raises problem with data volume and processing [14]. Google Earth Engine (GEE), a free cloud-based platform, has includes basic capabilities for the sophisticated calculations required for geospatial studies on a global scale [15]. Currently, registered users can access GEE via the web-based GEE Explorer and Code Editor platforms. Users can examine restricted satellite imagery with GEE Explorer, but they can undertake analysis and customization by scripting (Python or JavaScript) codes with the GEE Code Editor. Users can utilize the mathematical and spatial operations available in the Code Editor environment to customize it, according to the research objectives. Compared to conventional remote sensing methods, the GEE has demonstrated significant promise for classifying land cover [15,16]. Along with various automated mapping techniques, it has also made available a sizable public data catalog that includes information on land cover, weather, topography, population, and remote sensing data (such as MODIS and Landsat images) [17]. GEE provides support for categorizing images in many ways, such as cloud masking, filter image collection, basic Landsat composites, and cloud scoring. Prediction models for land use and cover often aim to identify areas where changes have occurred or may occur [18]. GIS and RS evolved to improve change detection and database advancement. Satellite imagery now provides simulations, spatial and temporal dynamics, and LULC information. It also provides a reliable platform for photographing, organizing, and analyzing digitized data [19,20].

There are various approaches and techniques that can be used to predict and model land use and cover changes. For examples artificial intelligence networks (ANNs), mathematical models, multiagent-based models, land use and its effects modeling frameworks (ANNs), GEOMOD models and expert system models have all been used in recent years [[21], [22], [23], [24]]. Many researchers have applied CA-Markov (CA-MCM) and Markov Chain Models (MCM) to simulate and predict LULCC in different environments. For example, from central Florida, USA [25], applied spatio-temporal data to study the application of Cellular Automata and Markov models to predict changes in the Saddle Creek Drainage Basin [26]. MCM and cellular automata (CA) were used to anticipate future land usage in the research regions [27]. The pivotal phase in the CA-MCM revolves around the transition rules, which are contingent upon the training data [28]. Moreover, the effectiveness of the applied model is influenced by factors such as the neighborhood class and cell size, both of which play a crucial role in obtaining ideal results from simulation or prediction [27]. The hybrid CA-MCM can efficiently use GIS and remotely sensing data and can transform results into spatially precise results, required for LULC mapping [29].

Afghanistan's predominant LULC categories are arable land, grassland, and forested regions. Arable land takes up only 12 % of the total land area, and less than 6 % of it is now under cultivation. Approximately 45 % of the country is made up of grasslands, whilst only 2 % is made up of forests. Afghanistan is regarded as one of the poorest countries in the world, with more than 80 % of its population depends on agriculture for a living. Agricultural and related activities account for more than 40 % of the total labor force. As such, ensuring a sustainable supply of ecosystem services is imperative for the country's sustainable development [30,31].

There is a gap in the current body of literature for historical and future simulations of LULC. Very few studies [[31], [32], [33]] have been undertaken to investigate LULC in Afghanistan and in the KRB. The existing studies have mainly focused on the current state of LULC, leaving a void in our understanding of the long term historical, future trajectory of these dynamics and social drivers within the basin. A thorough investigation into the simulation of potential LULC scenarios is required for well-informed decision making and the long-term management of natural resources in the KRB, considers the impact of unforeseen events and future land use changes.

Detailed knowledge of LULCC is crucial due to KRB's transboundary nature and its significance as a region confronting ever-growing urbanization and environmental problems. The dynamic nature of urbanization, coupled with the adverse impacts of climate change, poses significant threats to food security and agricultural stability in the region. Over the next 30 years, these changes are expected to intensify, with rising temperatures and unpredictable rainfall patterns leading to frequent droughts and floods. These environmental stressors not only damage crops and agricultural infrastructure but also exacerbate the strain on the basin's resources due to a drastically increasing population.

To address these pressing issues, our study aimed to employ GEE and the CA-MCM to conduct a long-term investigation of LULC changes within the Kabul River Basin. By integrating this advanced modeling approach, we sought to provide detailed future projections of LULC, specifically focusing on the spatial distribution and expansion of built-up areas. The study generated future LULC maps and statistical insights that are crucial for understanding the trajectory of urbanization and its implications on the environment and food security.

In this innovation, the integration of CA-MCM with geospatial techniques marks the first attempt to map and predict long term LULC for the entire basin. The application of the CA-MCM and geospatial techniques are expected to yield valuable insights into the spatial dynamics of urban growth. This information is essential for policymakers, urban planners, and environmental managers to devise strategies that mitigate the adverse effects of urban expansion and climate variability. Ultimately, this study aimed to contribute to the sustainable development of the KRB by providing a robust scientific basis for managing LULC changes and enhancing resilience against climate-induced challenges.

The primary objectives of the study area as under.

• To investigate the long term LULC changes from 1990 to 2020, using the Landsat images in Kabul River Basin
• To evaluate the future prediction of LULC for the years 2030, 2040 and 2050, using a Markov model.
• To identify key variables affecting LULC in the Kabul River Basin

## 2 Materials and methodology

### 2.1 Study area

**Fig. 1:**
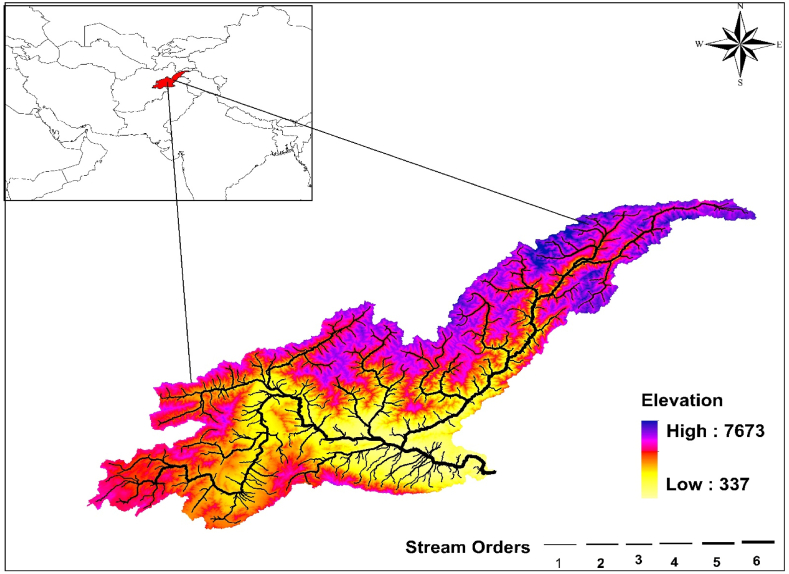
Kabul River Basin with its location, and topography.

The Kabul River Basin (KRB) has a rugged terrain and diverse topography, spans from 33.620 to 36.50 Latitude and. 67.560–73.8 Longitude which is shown in (Fig. 1). Based on the central statistical organization (CSO) data for 2014–15, the population density of the Basin is expected to be approximately 138 people per km^2^. The catchment has an area of 68,100 Km^2^ and is situated in eastern part of Afghanistan [34]. Elevation above sea level varies from 380 m on the eastern border with Pakistan (downstream) to 7700 m in the northern mountains (upstream). The basin's climate is semi-arid, and receiving and average precipitation 690 mm annually [35]. The Kabul River originates in the Hindu Kush Mountains and flows 700 km before entering the Indus River.

The KRB's notable climatic variability within its sub-basins, coupled with its semi-arid and strongly continental climate, significantly impacts each region's hydrological and physiographic features [36]. The six sub-basins (Table 1) than make up the KRB are Chitral, Kunar, Laghman, Logar, Kabul, and Panjsher. These sub-basins are named after their distinct climatic, hydrological, and physiographic characteristics. Other natural components that contribute to distinction include glacier position, snowmelt amounts, and seasonal precipitation patterns [37]. Table 1 shows the characteristics for each sub-river basin.

**Table 1:** Details of sub-river basins in KRB.

**#**	**Sub Basin**	**Annual Precipitation (mm)**	**Area (Km**^**2**^**)**	**Temperature (°C)**			**Mean Elevation (m)**		
				**Average**	**Max**	**Min**	**Average**	**Max**	**Min**
**1**	Chitral	680	14,610	2.1	14	−22	4020	7701	1065
**2**	Panjsher	794	12,951	5.7	20	−12	2898	5694	1032
**3**	Logar	481	9957	7.5	25	−10	2729	4296	1763
**4**	Laghman	719	6237	9.1	28	−8	2864	5432	639
**5**	Kunar	821	11,613	8.3	35	−6	2864	6229	492
**6**	Kabul	511	12,731	14.4	32	−5	1916	4692	380

### 2.2 Data set

#### 2.2.1 Data preparation

Several research have used the Google Earth Engine platform to explore long-term trends in land use and coverage [[38], [39], [40]]. Landsat datasets from the United States Geological Survey (USGS) which are accessible at https://www.usgs.gov can be obtained through Google Earth Engine [41]. It's the ideal platform for large-scale geospatial agricultural and environmental modeling because of its abundant reusable libraries, powerful computational capacity, variety of geographic datasets, and intuitive Application Programming Interfaces (API) [42]. To cover the whole study area, on average 15 Landsat images/tiles were obtained from 1990 to 2020 with five-years intervals for KRB (Table 2) by using GEE platform. The following steps were carried out during processing the required images. Landsat images collection was carried out by using Java Script in GEE and obtained images with cloud cover less than 2 % for ensuring the quality of the images. To further enhance the image quality, the built-in cloud masking function in GEE was applied. This step is crucial for eliminating any remaining cloud cover that might affect the accuracy of the classification. The selected images were composited to create a median image for each year, reducing the impact of any remaining clouds and anomalies. This composite represents the median reflectance values, providing a clear and consistent image for classification. The time cost of processing remote sensing images in GEE can vary depending on several factors, including the complexity of the preprocessing steps, the size of the study area, and the server load. In this study, the processing time included, the initial filtering and collection of images generally took a few minutes due to high computing power of GEE. The application of GEE's cloud-based infrastructure significantly reduces the processing time as compared to the traditional desktop GIS and software.

**Table 2:** Detail of the Landsat imageries.

**S.#**	**Year**	**Type of Satellite**	**Sensor**	**Date of acquisition**	**Cloud Cover (%)**	**Remarks**
**1**	1990	Land-sat 5	TM	May–Sep 1990	1.5	Target Months, minimum cloud covers
**2**	1995	Land-sat 5	TM	May–Sep 1995	1.5	
**3**	2000	Land-sat 7	ETM+	May–Sep 2000	1.5	
**4**	2008	Land-sat 5	TM	May–Sep 2008	2.0	
**5**	2010	Land-sat 5	TM	May–Sep 2010	1.5	
**6**	2015	Land-sat 8	OLI/TIRS	May–Sep 2015	1.5	
**7**	2020	Land-sat 8	OLI/TIRS	May–Sep 2020	1.5	

The JavaScript application programming interface (API) was utilized for preprocessing, mosaicking, clipping, and processing the obtained images. However, 2008 images were used rather than 2005 images because of technical problems (scan line error) with satellite data from 2003 to 2007 over the research areas for LULC change assessment. We used top-of-atmosphere (TOA) reflectance products from Operational Land Imager (OLI) Collection 1 Tier 1 and Landsat Thematic Mapper (TM) for this study. The technique used by TOA reflectance was chosen because it eliminates exoplanetary effects caused by fluctuating solar irradiation. This algorithm considers the solar zenith angles, spectral band variations, and Earth-to-Sun distances at different times of the year [43]. The aim was to capture the prevalent vegetation, minimize variations in ground reflectance, and reduce atmospheric haze between the months of May through September [44]. The slope, elevation, and aspect data were obtained from a digital elevation model (Dem) with a spatial resolution of 30 m. Both the DEM and Landsat images were sourced from GEE. Spatial variables such as distance from roads, rivers, water bodies and population density were obtained from Diva GIS and used for future LULC prediction.

#### 2.2.2 Satellite image processing

The GEE platform (https://earthengine.google.com) was the only option for processing Landsat TM and OLI data. GEE offers a quick study based on Google's computing infrastructure, which gives online datasets very instantly. Landsat 5 (1990, 1995, and 2008), Landsat 7 (2000 and 2010), and Landsat 8 (2015 and 2020) surface reflectance images were used to classify the research region's land cover and mentioned in (Table 2). To reduce the effects of clouds and cloud shadows, the required images were selected and mosaicked with the quality evaluation band flags. The Landsat image's spectral bands, specifically the red, green, and blue (RGB), near-infrared (NIR), and short-wave infrared (SWIR) bands, were chosen for classification in this manner. All image processing was carried out with GEE Code Editor (https://code.earthengine.google.com).

### 2.3 Random Forest Classifier

A well suited nonparametric, machine learning technique, Random Forest Classifier (RF) was used for mapping LULC. It is suitable for both regression and classification tasks. In addition, this classifier produces better classification accuracy [45]. When compared to other current algorithms, its usage in remote sensing offers various advantages, one of which is the high accuracy of the mapping of land use/land cover [46]. When there is a skewed class size distribution, the RF approach balances the classification error [47].

When operating, the RF method demonstrates rapid processing times [48]. The pixel-based execution technique of GEE's RF was chosen due to its robustness to data noise and overfitting. Its low sensitivity to overfitting makes it suitable for identifying satellite data [49,50]. Each decision tree comprises numerous nodes, and the results are determined by the participants' majority vote. This classifier can handle hundreds of input variables and potentially provide very accurate results [51]. Breinam (2001) claims that RF has numerous classifiers that are defined by Equation (1).

(1) $$\{\text{DT}\;\left(y,\sigma i\;\sum_{i=1}T\right)\}$$

where DT, is the decision tree, y is the input vector and σi, is a randomly sampled vector that has the same distribution as the σi, …, σi – 1 previous vector. Training data is provided by T boost strap. A regression tree is constructed for each bootstrap sample β using only one of M (attributes) randomly selected attributes chosen for the split at each point of the CART. Every bootstrap sample produces a no-pruned classification. By stabilizing the classifier, the RF increases the LULC classification performance and is more resistant to small changes in the input data. I equal-sized samples are retrieved via bootstrap selection from the training sample set. I tree were made for every sample, yielding i classification outcomes. By using each record to categorize with precision, the final LULC classification was established. The RF classifier uses object-based processing techniques to improve classification accuracy [51]. Key hyperparameters in the RF classifier include the number of trees (estimators), the maximum depth of the trees (max_depth), the minimum number of samples required to split a node (min_samples_split), and the minimum number of samples required to be at a leaf node (min_samples_leaf).

Based on the previous studies [52,53] as well as pretests that were conducted with our own data, we used 100 trees (ntree = 100) and the default for mtry, which equals to the square root of the total number of attributes.

### 2.4 LULC classification

For LULC classification, data from Landsat 5 (1990, 1995 and 2008), Landsat 7 (2000 and 2010), and Landsat 8 (2015 and 2020) have been used. The following five major classes (Table 3) were selected for investigation purposes: waterbodies, vegetation, snow cover, bare land, and built-up areas. A total of 1100 samples were selected for supervised classification in each target year for five different LULC classes. The number of samples per LULC class depends on the extent of LULC class. The dataset was split into training and validation sets with a 70:30 ratio. LULC is categorized by using Java Scripts API in GEE. The following three major steps were used through GEE for LULC classification in the KRB.

• Landsat image acquisition for target years
• Collection of training samples
• Selection and running of classifier.

**Table 3:** The description of land cover classes in the Kabul River Basin.

**#**	**Feature Class**	**Description**
**1**	Waterbodies	This class includes lakes, ponds, rivers, canals, streams, dams
**2**	Vegetation	Forest, planted or natural forest, agriculture crops, evergreen plants, and mix forest lands
**3**	Snowcover	Snowcover area
**4**	Bareland	Fallow land, exposed rock, strip mines, quarries,
**5**	Builtup	Builtup areas of all types of settlement, industrial areas, roads, and other artificial surface

### 2.5 Accuracy assessment

Without model evaluation, the machine learning process is not considered reliable [54]. Following the LULC categorization, the accuracy of the applied machine learning was examined to determine the models' performance. Using JavaScript, the data points related to waterbodies, snow cover, bare land, and built-up areas have been divided into 70 % training and 30 % testing datasets. The GEE-built Confusion Matrix (CM) is essential in mapping and validation process [55]. It compares the ground reference with the expected class label. The CM can be used to compute accuracy metrics like User Accuracy (UA), Kappa Co-efficient (Ka), and Overall Accuracy (OA) [56]. For every class, the scores for Kappa Co-efficient, UA, and PA were determined. The CM values and the Kappa Coefficient were used to assess the classification's performance. By employing RF's CM, the accuracy of the of different LULC classes were evaluated [57]. The required equations which are used in the literature [[58], [59], [60]]. equations (2), (3), (4), (5) represent the overall accuracy, producer accuracy, consumer accuracy and kappa coefficient respectively.

(2) $$\text{Overall}\;\text{Accuracy}=\frac{\text{Number}\;\text{of}\;\text{correctly}\;\text{classified}\;\text{iamges}\;}{\text{Number}\;\text{of}\;\text{total}\;\text{samples}\;}$$

(3) $$\text{Producer}\;\text{Accuracy}=\frac{\text{Number}\;\text{of}\;\text{correctly}\;\text{classified}\;\text{sample}\;\text{in}\;\text{each}\;\text{class}\;}{\text{Number}\;\text{of}\;\text{sample}\;\text{classified}\;\text{to}\;\text{that}\;\text{class}}$$

(4) $$\text{Consumer}\;\text{Accuracy}=\frac{\text{Number}\;\text{of}\;\text{correctly}\;\text{classified}\;\text{sample}\;\text{in}\;\text{each}\;\text{class}\;}{\text{Number}\;\text{of}\;\text{sample}\;\text{from}\;\text{refrence}\;\text{data}\;\text{in}\;\text{each}\;\text{class}}$$

(5) $$\text{Kappa}\;\text{Coefficient}=\frac{\text{Overall}\;\text{accuracy}-\text{estimated}\;\text{chance}\;\text{agreement}\;}{1-\text{estimated}\;\text{change}\;\text{agreement}\;}$$

### 2.6 Methodology framework for change detection and prediction

**Fig. 2:**
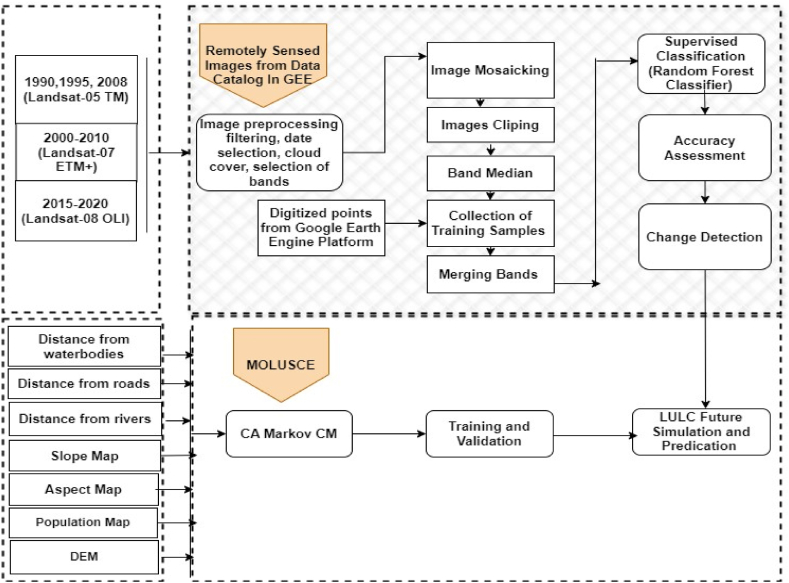
Methodology flow chart for LULC determination and LULC future projection.

The Quantum GIS (QGIS) version 2.18.10 software, with the assistance of Cellular (MOLUSCE) plugin and GEE were applied for the identification of both detection and predication of time series LULC changes. The analysis included the determination of changes in study area between the starting year (1990) and the final year (2020) of the LULC. By using the CA-Markov Chain Model (CA-MCM), LULC predication were made for the years 2030, 2040, and 2050 respectively. The major steps considered for this research are illustrated in the methodology flowchart, as depicted in (Fig. 2).

### 2.7 Cellular Automata Markov Chain Model (CA-MCM)

The Cellular Automata Markov Chain Model (CA-MCM) is a common method for simulating spatiotemporal changes in LULC systems. Both Markov Chain (MCM) a quantitative temporal model and Cellular Automata a spatial model used in the CA-MCM to predict LULC changing trends and characteristics over time. The MCM depicts the likelihood of cells transforming into different forms, but it lacks geographical distribution, and this justifies the integration of model. Thus, the MCM is hybridized with CA to distribute the expected changes spatially [[61], [62], [63]]. Two historical LULC maps serve as the primary inputs for the transition area matrix and transition probability matrix in the stochastic model MCM [62] and provide data on the number of cells expected to change LULC classes according to a transition probability matrix [64,65]. To project LULC change, MCM uses Bayes' Equation (6) as a basis, while Equation (7) is used to calculate probability matrix.

(6) $$S_{\left(t+1\right)}=P_{\text{mn}}\;*\;S\left(t\right)$$

(7) $$P_{\text{ij}}=\left\|\begin{array}{ccc} P11 & P12 & p1\ldots Pn1\\ P21 & P22 & P2\ldots Pn2\\ P31 & P32 & P33\ldots Pn3\\ . & . & .\\ . & . & .\\ . & . & .\\ Pm1 & Pm22 & Pm3\ldots Pmn \end{array}\right\|$$

where {0 ≤ P_ij_ ≤ 1 and $\sum_{m,n=1\;}^{j}(Pij)=1\;\left(m,n=1,2,3\ldots ,j\right)\}$ where P_ij_ denotes the transition probability matrix, i and j the number of LULC classes, at times (t) and (t+1), n is the number oof LULC classes categories and S(t) and S (t+1) are the statuses of LULC at time (t1) and t+1 respectively [9,66,67].

There is a spatial constraint that prevents MCM from identifying spatial variability in LULC. Thus, spatial and temporal LULC changes can be simulated by combining MCM with CA. CA can be expressed as Equation (8):

(8) S(t,t+1)=f{P(t),N}

where N is the number of Moore neighborhood cells (grids), ƒ is the transformation condition of the LULC classes, and S is the set of states of the finite cells at times (t) and (t + 1). It is noteworthy that CA is a dynamic process model utilized for LULC change. This type of model is rather common in the literature on LULC modeling. Because they are dynamic and reduplicate, each cell with its distinct qualities can represent both parcels of land and self-growth relationships. Furthermore, the condition of each cell is affected by the spatiotemporal states of its neighbors. Furthermore, the present condition and changes in neighboring cells can clarify LULC changes for each area and can mimic the expansion of objects in two directions. As a result, this model is often employed in geographic models for projecting future LULC.

The LULC projection was conducted in two steps by using two historical LULC maps (1) the Markov Chain Model was to produce transition probability matrix and transition area matrix (2) Then the developed matrices were put into the CA-Markov hybridized model to project or simulate the future LULC classes for the year 2020, 2030, 2040 and 2050 respectively and the model was set at 15 CA iterations. To check the accuracy of the LULC modeling, it was necessary to cross check the projected LUCL map for the year 2020 with the historical classified LULC map 2020 (reference map) and the accuracy was determined by using Kapa Co-efficient [68].

### 2.8 Future LULC prediction and validation

Following the LULC mapping from Landsat data over the period of 1990 and 2020 at 5-years intervals, future simulation of LULC change was conducted using the MLP-ANN approach in the MOLUSCE plugin included in QGIS software version 2.18.10. The CA-MCM is a comprehensive model that estimates the trends and geographic organization of LULC classes using historical LULC thematic maps, suitability matrices, and transition probability matrices [69,70]. Inputs were needed before moving on to the LULC projection. Firstly, three discrete LULC categorized maps were created; two of these maps were used to prepare the transition probability matrix, while the third map was used to validate the model. The geographical variables, which comprise the spatial and socioeconomic characteristics, were also used for LULC projection (Table 4).

**Table 4:** Driving parameters for LULC projection in Kabul River Basin.

**S.No**	**Parameter Type**	**Parameter**	**Source**
**1**	Socio-economic Parameters	Population Density	Diva GIS
**2**	Spatial Parameters	Elevation Map	SRTM
		Slope Map	SRTM
		Aspect Map	SRTM
		Distance from Roads	Diva GIS
		Distance from Rivers	
		Distance from Railways	
		Distance from Waterbodies	

Each LULC class's growth can be regulated by a number of factors, which can interact and form complex relationships. Major driving factors, such as topography, aspect, slope, and variables related to human disturbance, like roads, railroads, water bodies, and population density, were selected according to their accessibility and influence on LULC variations [[71], [72], [73]]. The appropriateness matrix was created using these motivating reasons. The classified LULC maps of the year 2000 and 2020 were used to generate a transition probability matrix and driving factors to create suitability matrix. Future LULC predictions can only be trusted if the simulations' results are verified against available datasets. Consequently, we employed the transition matrix generated from the thematic maps of the years 2000 and 2010 in conjunction with the driving variables (Table 4) to simulate LULC for the year 2020. A supervised classified LULC map for the year 2020, was used to validate this projected map of the 2020 [71,74]. After obtaining appropriate validation metrics, we used LULC data from maps from 2000, 2010, and 2020 to forecast future LULC in 2030, 2040, and 2050, respectively.

### 2.9 Correlation of population and LULC

The total population census for the study years 1990, 2000, 2007, 2017, 2018, 2019, and 2020, was obtained from the Afghanistan National Statistics Authority (NISA), World Bank previous published reports, and published research articles. The estimation of the inter-census annual population utilized the Inter-Census Annual Growth Rate (ICGR) formula (refer to eq (9)). To project or forecast population data from 2020 to 2050, Microsoft Excel's Exponential Smoothing Forecast tool was employed. This tool leverages time series historical population data (see Eq. (10)) and is based on the AAA version of the Exponential Triple Smoothing (ETS) algorithm. The ETS algorithm, a widely used statistical method for time-series forecasting, addresses minor deviations in past data trends by identifying seasonality patterns and establishing confidence intervals.

(9) ICGR = X + XR … …………………………………………………… …...…… ….………………… ….

where “R" is the growth rate factor recorded by the census, and “X" is the population from the previous year.

(10) Forecast = FORECAST.ETS (target year, values, timeline, [seasonality], [data completion], [aggregation]

## 3 Results

### 3.1 Spatio-temporal changes in the historical LULC

**Fig. 3:**
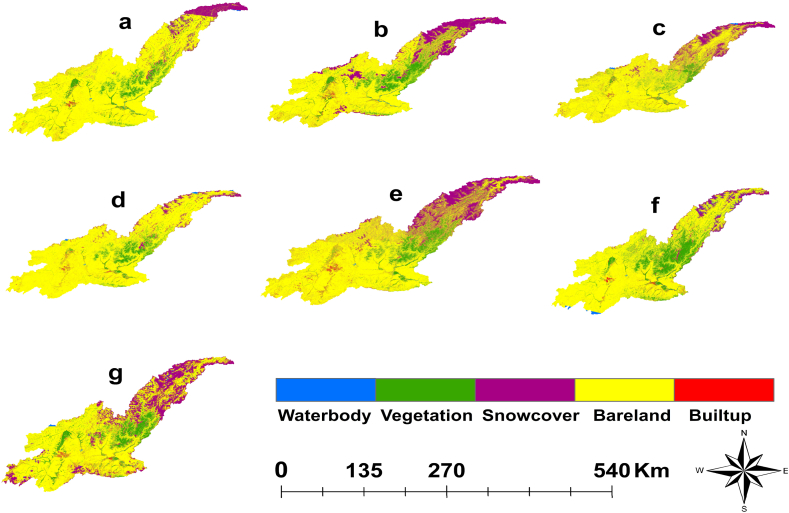
Classified (LULC) maps of KRB for the years 1990 (a), 1995 (b), 2000 (c), 2008 (d), 2010 (e), 2015 (f) and 2020 (g). Generated through the utilization of multi-date Landsat imagery and the random forest classification algorithm.

**Fig. 4:**
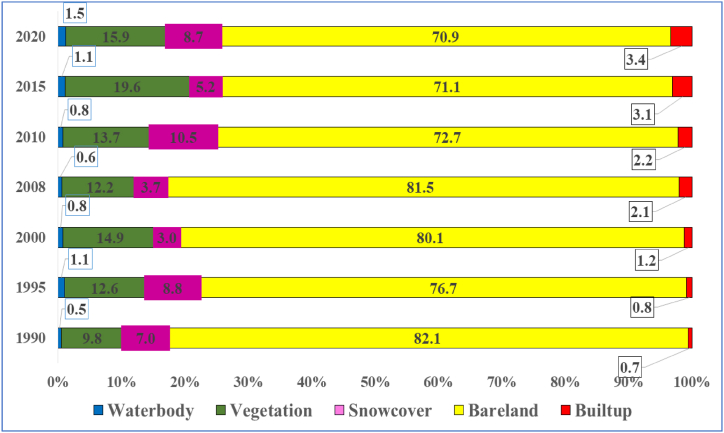
Quantitative temporal change assessment of LULC in KRB.

Five LULC classes (Fig. 3) were generated by using RF Classifier in the Google Earth Engine. Initially, it was perceived that there was an increasing anthropogenic activity in the study area due to urbanization and intensified agriculture practices. The classified LULC maps under each class category for the years 1990, 1995, 2000, 2008, 2010, 2015, and 2020 are represented by a, b, c, d, e, f and g respectively in (Fig. 3) and the related area statistics under each class category for the stated years are presented in (Fig. 4). Among the LULC features, the bare land was the most dominant LULC class in all the years occupying 70606 km^2^ (82.1 %) in 1990 and gradually decreasing to 48212 km^2^ (70.9 %) in 2020 followed by vegetation which exhibited variation periods but shows an overall increasing trend during the study period. The remaining feature classes depict increases and decreases over the study years, reflecting significant fluctuation which need further investigation.

The waterbody class occupied less than 2 % of the study's landscape as depicted (Fig. 4) and shows variation during the study period. However, the study determined that there were significant changes in LULC in the KRB over time, with the growth of built-up areas noticeably increasing. The built-up area drastically increased from 476 km^2^ (0.7 %) in 1990–2312 km^2^ (3.4 %) in 2020. But the vegetation area exhibited variation, occupying its lowest point in 1990 amounting to 6528 km^2^ (9.8 %) and then increasing to 10812 km^2^ (15.9 %) in 2020. Snowcover and waterbody areas showed fluctuation over the years, possibly influenced by climatic factors. However, between 1990 and 2020, the built-up class significantly increased, mostly due to the conversion of bare lands into built-up areas.

### 3.2 Accuracy assessment of classified LULC

Based on the Confusion Matrix (CM), the accuracy evaluation metrics like Overall Accuracy (OA), Producer Accuracy (PA) and Consumer Accuracy (CA) of the classified LULC maps were generated (Table 5). In general, all the classified LULC Maps produced high accuracies (OA ranges from 83 % to 95 %). The CA in the various classes has increased from 84.5 % to 94.0 % and similarly, the PA has risen from 82.0 % to 92.0 % during 1990–2020. The OA for the various LULC classes shows an increasing trend from 1990 to 2020, this could be linked with the improvement in the digital visualization of satellite images. The Kappa Coefficient of the classified images ranges from 0.81 % to 0.92 % and falls in the range of acceptable levels.

**Table 5:** Statistics of classified images in KRB.

**Year**	**CA (%)**	**PA (%)**	**OA**	**Kc**
**1990**	84.50	82.00	83.00	0.81
**1995**	85.00	83.00	88.00	0.82
**2000**	88.00	86.00	87.00	0.84
**2008**	95.00	91.00	93.00	0.90
**2010**	94.00	91.00	92.00	0.90
**2015**	95.25	93.00	95.00	0.92
**2020**	94.00	92.00	93.95	0.91

### 3.3 Spatial change detection and LULC transitions mapping

**Fig. 5:**
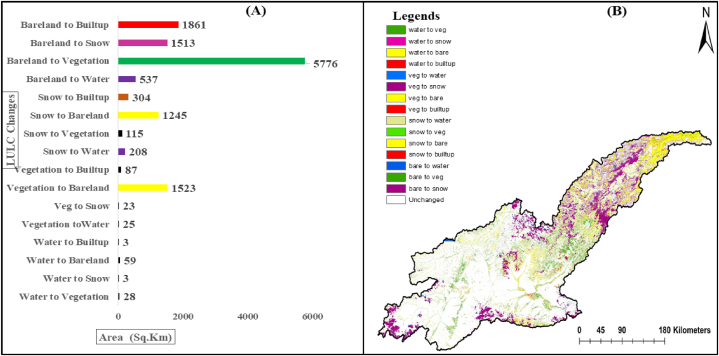
Spatial change statistics (A) and change detection map (B) of Kabul River Basin from 1990 to 2020.

The results in (Fig. 5) shows the transition of each of the five LULC classes during 1990–2020. During the study period (1990–2020), the four main LULC transitions occurred, these were snow to bare land, bare land to vegetation, bare land to built-up area and vegetation to bare land. The spatial change was determined to quantify and visualize the transition of one LULC feature to another from 1990 to 2020.

Both visually and quantitively, the main changes and transformations occurred are represented by (A) and (B) in (Fig. 5) respectively. Based on the study results, substantial interclasses transformation occurred. The prominent and significant transformation and conversion occurred in the bare land to vegetation, followed by vegetation to bare land, snow cover to bare land, and bare land to built-up area, respectively. The results show that 5776 Km^2^, 1861 km^2,^ and 1513 km^2^ areas of bare land have been transformed into vegetation, built-up and snow-cover respectively. Similarly, 1745 km^2^ and 1023 km^2^ areas of snow cover and vegetation have been converted to bare land respectively. The built-up area has drastically increased by over 400 % during the study period.

### 3.4 Future prediction of LULC

**Fig. 6:**
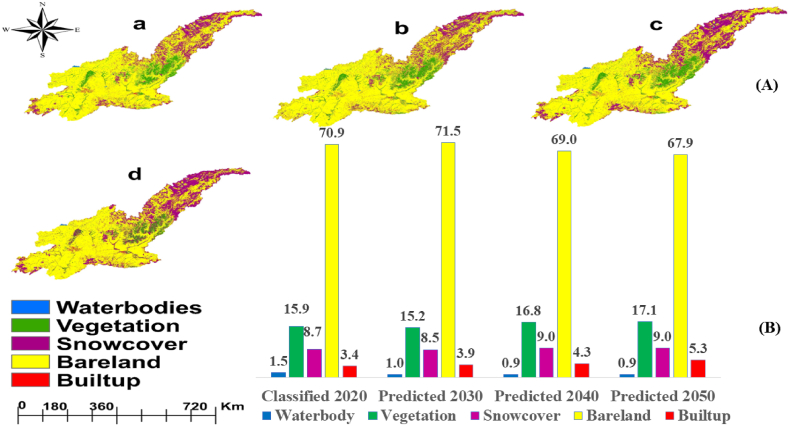
Predicted LULC maps (A) for the years 2020 (a), 2030 (b), and 2040 (c) and 20250 (d). Predicted LULC statistics (B) for the years 2020, 2030, 2040 and 2050 respectively.

CA-MCM built in QGIS was used in this study to project future LULC for the KRB for the years 2030, 2040, and 2050 Fig. 6. The pictorial comparison in (Fig. 6) are represented by a, b, c and d of the classified and projected LULC Maps, shows that, CA-MCM effectively projected LULC Maps for KRB. In 2020 and 2050, the model projected a significant decrease in bare land area amounting 48212 Km^2^ (70.9 %) to 46172 (67.9 %) and increase in built-up area amounting 2312 Km^2^ (3.7 %) 3640 Km^2^ to 5.5 %.

The model also predicted a slight increase in snow cover (7.9 %–9.0 %) and an increase in vegetation area from 10812 km^2^ (15.9 %) to 11628 km^2^ (17.1 %). Whereas waterbodies were projected to be consistent (1.2 %–0.9 %) between 2020 and 2050. The validation results demonstrated that the model effectively simulated the future LULC pattern in the Kabul River Basin by having overall correctness of 88 % and a kappa coefficient of 0.85 which is above the value of 0.75 [75,76]. This suggests an accurate model performance because the simulated LULC maps of the year 2020 were quite like the classified 2020 that was generated in this study. The classified and projected LULC maps for the year 2020 revealed that there are substantial variances and closeness in LULC classes, hence both agreements and differences existed between classifier and projection models. In 2020, the classified area for water bodies was 744.7 km^2^, while the area that was projected was 620.6 km^2^ which meant that there was a reduction of 124.0 km^2^ and this corresponds with a decrease of 16.6 %. This shows possible under estimation of water bodies in the forecast model. Similarly, vegetation class, classified as being 10812 km^2^ against one that was expected to be 11628 km^2^. The slight difference of 816 km^2^ which also represents 7.5 % points out that projection model closely calculated the amount of vegetation cover.

### 3.5 Correlation between population and LULC

**Fig. 7:**
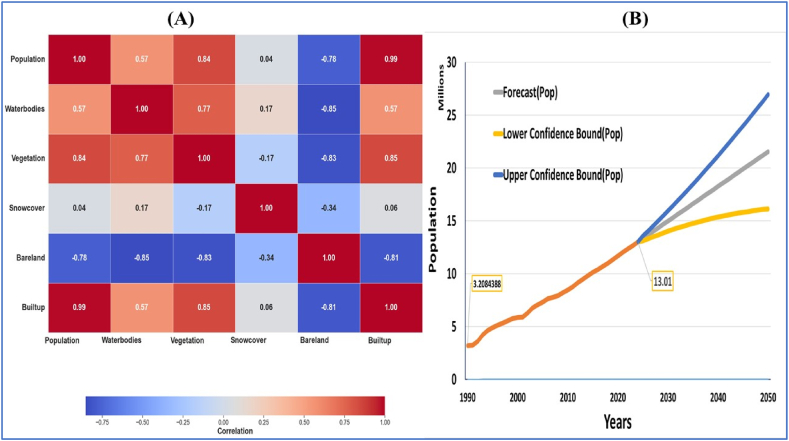
Correlation Matrix between population and LULC **(A).** Statistics of historical and projected population in Kabul River Basin **(B).**

The KRB's population and various LULC classifications' correlation represented by (A) and (B) are shown in (Fig. 7). The historical population data was obtained from Afghanistan National Statistics Authority (NSIA) and previous World Bank published reports. The annual population data was estimated from the growth rate given by the recorded population census. Future population projection for the year 2020–2050 was carried out by using Microsoft forecasting tools (Fig. 7). Both the historical and projected populated records show increasing trends in the study area. Projected population data was required to correlate land use land cover changes occurred after 2020. A strong positive and statistically significant correlations were observed between population and vegetation (r = 0.84, p < 0.001), representing a noteworthy increase in green cover with population growth. Similarly, a highly positive correlation was found between population and built-up areas (r = 0.98, p < 0.001), highlighting the close relationship between urbanization and population expansion. Conversely, a strong negative correlation was identified between population and bare land (r = −0.7793, p < 0.001), implying a significant reduction in undeveloped areas as the population increases. No significant correlation was found between population and snow cover (r = 0.0366, p = 0.8448), indicating that snow cover dynamics are not strongly influenced by population changes. The correlation matrix among LULC classes further reveals insights into the interrelationships within the basin. Vegetation and built-up areas exhibit a robust and positive correlation (r = 0.9886, p < 0.001), indicating a simultaneous increase in green cover and urban infrastructure. Conversely, the bareland demonstrates a strong negative correlation with both vegetation (r = −0.77, p < 0.001) and built-up areas (r = −0.94, p < 0.001), underscoring the transformation of undeveloped areas with urbanization. Snow cover, on the other hand, displays a weak correlation with other LULC classes, suggesting a limited influence of land use changes on snow cover patterns.

## 4 Discussion

This study utilized the integration of land modeling and geospatial techniques for the first time to investigate and predict long term LULC in KRB over the period from 1990 to 2020. Furthermore, for the KRB, this is the first attempt to assess the LULC rate of change, primary causes, intensity, and transition across a 30-year period (1999–2020).

Historical investigation of LULC and their future prediction are essential for knowing the current trend of natural resources utilization and sustainable land management. GEE and land modeling techniques are commonly used for LULC assessment. Our results indicate that bare land is the most dominant class and amounting from 70606 km^2^ (82.1 %) to 48212 km^2^ (70.9 %) over the study period from 1990 to 2020. Our findings are aligned with the results of Hussain et al. [77]. Rugged topography, steep slope, narrow valleys, high mountains and low precipitation (400 mm) that characterize the Kabul River Basin, make it bare and fallow [78]. Our results also depicted drastic LULC change between 1990 and 2020, with an increase in vegetation and built-up area, and a decrease in bare land areas. Anthropogenic activities, like the horizontal growth of urbanization and agricultural regions by residents as well as investors and other communities from nearby provinces and nations, may be the cause of these land use dynamics.

According to our study, builtup area has increased drastically from 476 km^2^ (0.7 %) to 2312 km^2^ (3.4) % during 1990 and 2020. This analysis indicates substantial changes over the study period. It is worth noting that there has been a significant overall increase in the built-up area from 1990 to 2020, amounting to 1836 km^2^ (2.74 %). This has been confirmed transition of bare land to built-up area, specifically in low and plains area and in high mountain fertile valleys. Commonly, the decrease in the bare land area could be linked to urbanization and agriculture expansion. However, the expansion in agriculture is mostly related to a substantial increase in population and food demand. This expansion in built-up area mainly occurred in central and low plain areas where the capital city and provincial cities are located in KRB.

The pronounced expansion in the built-up area can be traced back to pivotal historical events, specifically the overthrowing of the De-Facto regime in 2001. The establishment of the new government, the Islamic Republic of Afghanistan, marked a critical stage in this political transition. The subsequent increase in local and international investments, repatriation of million Afghan refugees from neighboring countries and internally displaced people played a pivotal role in the increase of urbanization. The shift in governance and increased stability in the region facilitated an environment conducive to substantial infrastructure projects and housing schemes, thereby fueling built-up areas and settlements within the KRB.

As one of the most densely inhabited river basins in Afghanistan, the study area's expansion is primarily owing to the region's expanding industrial districts and population [78]. Alongside this, there is a rise in vegetation, which makes sense considering the growing population's demand for food.

The study reveals that there hasn't been much change in the water class over the study period. The water class percentage, which ranges from 340 km^2^ (0.5 %) to 816 km^2^ (1.2 %), indicates a comparatively stable state. The water coverage values that have been observed, although they fall within this small range, demonstrate a noteworthy consistency in the spatial distribution of water resource in the study area. There are a few important reasons why the water class appears to have not changed significantly. The stability that has been observed may primarily be attributed to the low degree of development of the water resources infrastructure and the dearth of water reservoir and irrigation canal restoration projects within the KRB. The absence of large-scale projects involving the development of water resources may limit the likelihood of notable changes in the water class. This emphasizes the requirement for integrated strategies for the development of water resource and of water reservoir projects for addressing the potential concerns about a shortage of water and enhance the resilience of the water class within the basin.

The Overall Accuracy (OA) ranged from 83 % to 93.5 % for images from 1990 to 2020. This is greater than the recommended LULC categorization accuracy of 70 % [79,80]. However, the progress improvement in our LULC classification accuracy from the initial study year 1990 to the final year 2020 could depict challenges linked with the availability of older Google Earth images from prior decades with slightly greater spatial resolution.

The future prediction of LULC is essential for sustainable natural resources management and planning. Recently several techniques have been put in place for land modeling such as Artificial Neuro Network, mathematical models, multiagent-based models, land use and its effects modeling frameworks (ANNs), GEOMOD models and expert system models. The CA-Markov Chain Model is an effective tool and widely used one for projection of LULC [8,9]. This method is based on historical data to project the probability of future scenarios of LULC. Simplicity, accuracy and efficiency are the main advantages of this method [8]. Our results indicate that the model effectively projected LULC for the years 2030, 2040 and 2050 in the study area. According to our findings bare land would decrease from 48212 km^2^ (70.9 %) to 46172 km^2^ (67.6 %) from 2020 to 2050. This could be associated with meeting the shelter and food demand of the increased population in the KRB where the population mainly depends on agriculture and farming activities for their livelihood. Similarly, the built-up area would increase from 2312 km^2^ (3.4 %) to 3640 km^2^ (5.3 %) from 2020 to 2050 and the similar trend was presented by Hussain et al. [9].

Projection of LULC can help policy makers and planners in the identification of potential areas for climate change mitigations measures like identification of areas for afforestation, reforestation and greenhouse gases emission from LULC practices [9]. Significant environmental impacts such as changes in water availability, habitat loss and greenhouse gas emissions are associated with the LULC. Predication of these changes could help decision makers and researchers understand the possible impacts on the environment and formulate strategies to reduce adverse impacts. The impacts of LULC changes can also be felt in the social and economic systems which include crop yield variations, changes in water availability as well as displacements of people. Predication of these changes can help in the assessment of potential risks and vulnerability and devise strategies to address them. The findings of the future projection of LULC changes can give useful information for environmental planners, decision-makers and other relevant stakeholders [51]. Our findings can help identify areas that are probably to experience substantial land use changes, evaluate and assess the potential impacts of those changes on the society and environment and evaluate the effectiveness of the various LULC policies and scenarios.

The CA-Markov Chain Model is an effective to project future LULC, but it has some limitations that need to be adjusted. Validation of the model is a key step which requires historical and independent dataset to ensure their applicability and reliability [9]. Future study could focus on evaluating existing models in various places and circumstances to assess their performance and discover areas for improvement.

The results of the study show a significant positive association (r = 0.84, p < 0.001) between the increase in population and the spread of vegetation. This correlation suggests that as the population of the study area increases, there is a notable rise in the extent of vegetation. This result might be a result of afforestation efforts, urban greening programs, or other causes enhancing the amount of green space in response to population expansion. A remarkable positive association (r = 0.98, p < 0.001) has been seen between the rise in population and the expansion of built-up areas. The close association between population growth and urbanization in the study area is highlighted by this strong correlation. Infrastructure that is put up has grown significantly in tandem with population increase, indicating the effects of urbanization on the landscape.

There is a strong negative correlation (r = −0.78, p < 0.001) between the amount of bareland and population growth. The substantial negative correlation indicates that there is a large decline of bareland as population grows. This phenomenon may be explained by a decrease in open or undeveloped regions as a result of increased land development. There is a moderately favorable link (r = 0.57, p = 0.0008) between the number of water bodies and population growth. This correlation implies that as the population increases, there is a moderate increase in the extent of water bodies. This may be influenced by factors such as urban expansion or water resource management initiatives. The analysis did not reveal a significant correlation (r = 0.036, p = 0.84) between population growth and snow cover. This suggests that population dynamics may not be a key driver influencing snow cover in the KRB.

The findings of this study may be helpful to many stakeholders and policymakers, serving as a guide for them as they develop and manage the natural resources in the KRB. Additionally, the study offers some insights into the major drivers that may be extremely essential in modifying the landscape structure of the most significant and densely populated River Basin in the future. Informed urban planning, management of natural resources, and monitoring of grassland productivity could be made possible by understanding the areal size, change rate, intensity, and transition of key LULC categories such as bare land, built-up areas, vegetation, snow cover, and water bodies during a 30-year period. The findings of this study may be broadly applied to other land use and environmental planning initiatives in the KRB and maybe across Afghanistan.

## 5 Conclusions and recommendations

The primary objective of this study to map the long-term LULC changes, using multi-date Landsat imageries for the years 1990, 1995, 2000, 2005, 2010, 2015 and 2020 and then project the LULC changes years 2030, 2040 and 2050, using a Markov Chain Model in KRB. It is noteworthy that there has been a significant overall increase in built-up area from 1990 to 2020 amounting to 1836 km^2^ (2.7 %). Contrary to built-up area, the bare land has decreased drastically, amounting 70606 km^2^ (82.1 %) in 1990–48212 km^2^ (70.9 %) in 2020. According to CA-Markov Model's prediction, vegetation area would increase from 9618 Km^2^ (15.9 %) in 2020–11748 km^2^ (17.1 %) in the study domain. Our results showed that the built-up area would increase from 2020 to 2050, amounting to 1328 km^2^ (1.9 %). The vegetation lands are expected to increase, as well as bare land will be converted to built-up and agriculture land. There was a strong positive correlation between population and vegetation, as well as population and built-up areas. In contrast, a robust negative correlation exists between population and bareland, while no significant correlation was found with snowcover.

Monitoring and identifying LULC changes are crucial, as it would help national and global policymakers and planners to shape future environmental management. Proactive efforts are required to develop a more resilient and sustainable future. By recognizing the complex relationship between LULC and incorporating climate-conscious policies into land use planning, we can seek to mitigate the negative consequences of urbanization and climate change while protecting the integrity of our natural resources. Collaboration among stakeholders such governmental agencies, communities and non-governmental organizations will be critical in designing a more habitable and climate-resilient environment for future generations.

Future land-use land-cover prediction is an important field of study with wide implications for sustainable development, land use planning, and environmental management. Collaboration among governmental agencies, urban planners, and environmental stakeholders is pivotal to developing strategies and policies that enhance sustainable urbanization, address infrastructure needs, and safeguard the ecological health of the study area. The CA-Markov approach is an effective tool for anticipating LULC changes, although it does have some limitations that must be considered. A multidisciplinary approach can produce more precise and trustworthy estimates of LULC changes, allowing decision-makers to plan for a sustainable future. Finally, future study on LULC changes should look into how land use and land cover are affected by climate change, how land use regulations influence LULC changes, and how land use and biodiversity conservation interact.

## Funding information

This study was funded by Higher Education Commission of Pakistan under Allama Iqbal Scholarship for Afghan Students.

## Data availability statement

Data included in article/supp. material/referenced in article.

The data associated with this study has not been deposited in a publicly available repository because all relevant data is included within the article in the form of table and figures. The data can be fully reviewed and assessed through the provided figures and tables.

## CRediT authorship contribution statement

**Rahmatullah Wahdatyar:** Writing – original draft, Visualization, Methodology, Investigation, Formal analysis, Data curation. **Muhammad Fahim Khokhar:** Writing – review & editing, Writing – original draft, Visualization, Validation, Methodology, Formal analysis, Data curation, Conceptualization. **Shakil Ahmad:** Visualization, Supervision, Conceptualization. **Mohammad Uzair Rahil:** Validation, Data curation, Conceptualization. **Mohammad Ajmal Stanikzai:** Visualization, Data curation, Conceptualization. **Junaid Aziz Khan:** Visualization, Software, Methodology. **Kamran:** Visualization, Validation, Methodology.

## Declaration of competing interest

The authors declare that they have no known competing financial interests or personal relationships that could have appeared to influence the work reported in this paper.
